# N_2_O strongly prevents adhesion formation and postoperative pain in open surgery through a drug-like effect

**DOI:** 10.1186/s10397-017-1024-2

**Published:** 2017-11-07

**Authors:** Roberta Corona, Maria Mercedes Binda, Leila Adamyan, Victor Gomel, Philippe R. Koninckx

**Affiliations:** 1 Department of Obstetrics and Gynaecology, KU Leuven – Catholic University of Leuven, 3000 Leuven, Belgium; 2 Barbados Fertility Centre, Seaston House, Hastings, Barbados; 3 Department of Reproductive Medicine and Surgery, Moscow State University of Medicine and Dentistry, Moscow, Russia; 4 Department of Obstetrics and Gynecology, University of British Columbia, Women’s Hospital, Vancouver, British Columbia Canada; 5 KU Leuven, Vuilenbosstraat 2, 3360 Bierbeek, Belgium

**Keywords:** Postoperative adhesions, N_2_O, Conditioning, Humidification, Microsurgery, Microsurgical principle

## Abstract

**Background:**

Microsurgical tenets and peritoneal conditioning during laparoscopic surgery (LS) decrease postoperative adhesions and pain. For a trial in human, the strong beneficial effects of N_2_O needed to be confirmed in open surgery (OS).

**Results:**

In a mouse model for OS, the effect of the gas environment upon adhesions was evaluated. Experiment I evaluated desiccation and the duration of exposure to CO_2_, N_2_O or CO_2_ + 4%O_2_. Experiment II evaluated the dose-response curve of adding N_2_O to CO_2_. Experiment III compared humidified CO_2_ + 10% N_2_O during LS and OS.

In OS, 30- and 60-min exposure to non-humidified CO_2_ caused mortality of 33 and 100%, respectively. Mortality was prevented by humidification, by dry N_2_O or dry CO_2_ + 4%O_2_. Adhesions increased with the duration of exposure to CO_2_ (*p* < 0.0001) and decreased slightly by humidification or by the addition of 4% O_2_. N_2_O strongly decreased adhesions at concentrations of 5% or greater. With humidified CO_2_ + 10% N_2_O, adhesion formation was similar in OS and LS.

**Conclusions:**

The drug-like and strong beneficial effect of low concentrations of N_2_O is confirmed in OS.

## Background

The peritoneal cavity with its peritoneal fluid is a specific environment different from that of plasma. The mesothelial cell lining of the peritoneal cavity and its organs facilitates the gliding of the bowels and actively regulates homeostasis and transport of fluids, molecules and cells. In males, the volume of peritoneal fluid is small. In women of reproductive age, follicular exudation increases the volume and adds high concentrations of steroid hormones. The peritoneal cavity is not vascularised and constitutes a sterile cavity that does not belong to the body homeostasis. Any trauma in the peritoneal cavity causes an inflammatory reaction and a mesothelial cell retraction, exposing the basal membrane. This abolishes the blood-peritoneal fluid barrier and permits the entry of immunocompetent cells and facilitates diffusion of larger molecules as immunoglobins, which is an efficient defence mechanism to intruders [1, 2].

The large and flat mesothelial cells react within seconds to any trauma by retraction and bulging [1, 2] causing an acute inflammation [3] which increases with the duration and severity of the trauma. Identified traumas are surgical manipulation, mesothelial cell hypoxia by CO_2_ pneumoperitoneum, deeper ischaemia at an intraperitoneal pressure of more than 8 mmHg and ischaemia-reperfusion at desufflation [4], oxidative stress [5] or reactive oxygen species (ROS) induced by exposure to air with 20% of oxygen, desiccation and saline as irrigation liquid. The severity and the duration of this acute inflammation of the entire peritoneal cavity create an inversely proportional reduction of fibrinolysis. This, in turn, increases the potential of adhesion formation through a reduction in tissue plasminogen activator (tPA) and an increase in plasminogen activator inhibitor (PAI) [6, 7]. During laparoscopic surgery, the retraction and bulging of mesothelial cells cause a progressive increase in CO_2_ resorption. The acute peritoneal inflammation increases postoperative C-reactive protein concentrations (CRP) and causes postoperative pain [2].

During laparoscopic surgery, prevention of the mesothelial cell retraction and the subsequent acute inflammation effectively prevents or decreases the associated consequences including postoperative adhesion formation and postoperative pain. In addition, it accelerates recovery and in animal experiments decreases tumour metastasis. The most effective preventive factors are the addition of more than 5% of nitrous oxygen to the CO_2_ pneumoperitoneum, cooling of the peritoneal cavity below 31 °C, minimalising mechanical trauma and ROS production, using Ringer’s lactate instead of saline and administering one or two doses dexamethasone postoperatively [2]. If used together with a barrier [8], this approach results in virtually adhesion free surgery [9].

The similarity between our current knowledge derived largely from animal experiments, and the microsurgical tenets developed in the early 1970s empirically, but controlled by systematic second-look laparoscopy, 8–12 weeks after the initial operation, is striking. These principles were developed for open surgery and soon after applied in laparoscopic surgery [10]. These microsurgical principles indeed are a combination of gentle tissue handling, judicious use of electrical and/or laser energy, use of inert sutures, continuous irrigation with Ringer’s lactate at room temperature during the procedure to avoid desiccation, shielding the bowels from the ambient air, thorough lavage of the peritoneal cavity at the end of the procedure, instillation of Ringer’s lactate solution containing a minimum of 500 mg of hydrocortisone succinate into the peritoneal cavity before closure and administration of one or two doses of dexamethasone after surgery.

Microsurgical tenets were proven to decrease adhesion formation and to increase pregnancy rates in open and laparoscopic surgery [10, 11]. The relative importance of each of these factors that decrease acute inflammation and adhesion formation was investigated only recently in a laparoscopic mouse model with proof of concept trials in human [9]. However, the addition of low doses of N_2_O which is the single most effective factor was investigated during laparoscopic surgery with an insufflation pressure only. Since there is no insufflation pressure in open surgery, we, therefore, decided to evaluate the effect of N_2_O in a mouse model for open surgery before undertaking a trial in human.

## Methods

Animals and the experimental set-up (anaesthesia, ventilation, laparoscopic surgery, adhesion induction and scoring) were as previously described [3, 12, 13].

### Animals

Inbred 9 to 10-week-old female BALB/c OlaHsd mice of 18 to 20 g (Harlan Laboratories B.V., Venray, The Netherlands) mice were used to decrease experimental variability. They were kept under standard laboratory conditions and diet at the animal facilities of the Katholieke Universiteit Leuven (KUL). The study was approved by the Institutional Review Animal Care Committee (KUL: P040/2010).

### The laparoscopic mouse model

Following anaesthesia and pneumoperitoneum induction (Thermoflator, Karl Storz, Tüttlingen, Germany) with humidified gas (Humidifier, 204,320 33, Karl Storz) and standardised 10 × 1.6 mm bipolar lesions (20 W, standard coagulation mode, Autocon 350, Karl Storz, Tüttlingen, Germany) were made on both right and left uterine horns and on abdominal walls using a 2 mm endoscope (Karl Storz, Tüttlingen Germany) and two 14-gauge catheters (Insyte-W, Vialon, Becton Dickinson, Madrid, Spain) as secondary ports. The insufflation pressure was 15 mmHg. Since adhesion formation increases with body temperature, the latter was strictly controlled [13]. Therefore, mice and equipment were placed in a closed chamber at 37 °C (heated air, WarmTouch, Patient Warming System, model 5700, Mallinckrodt Medical, Hazelwood, MO). Anaesthesia and ventilation [14] and the timing between anaesthesia (*T*_0_), intubation (at 10 min, *T*_10_) and the onset of the experiment (at 20 min, *T*_20_) were standardised.

The only variable in this model of adhesion formation thus was the duration of the pneumoperitoneum and the type and humidification of the gas used.

### A mouse model for open surgery

All factors validated for the laparoscopic model were kept identical, i.e. animals, anaesthesia, intubation, ventilation, temperature control, timing, type of lesions, the equipment used for gas insufflation and humidification and the scoring of adhesions. The only difference was that instead of a laparoscopy with a pneumoperitoneum, a laparotomy was performed and the mice were kept with the open abdomen in a box exposed to the specific gas environment.

**Fig. 1:**
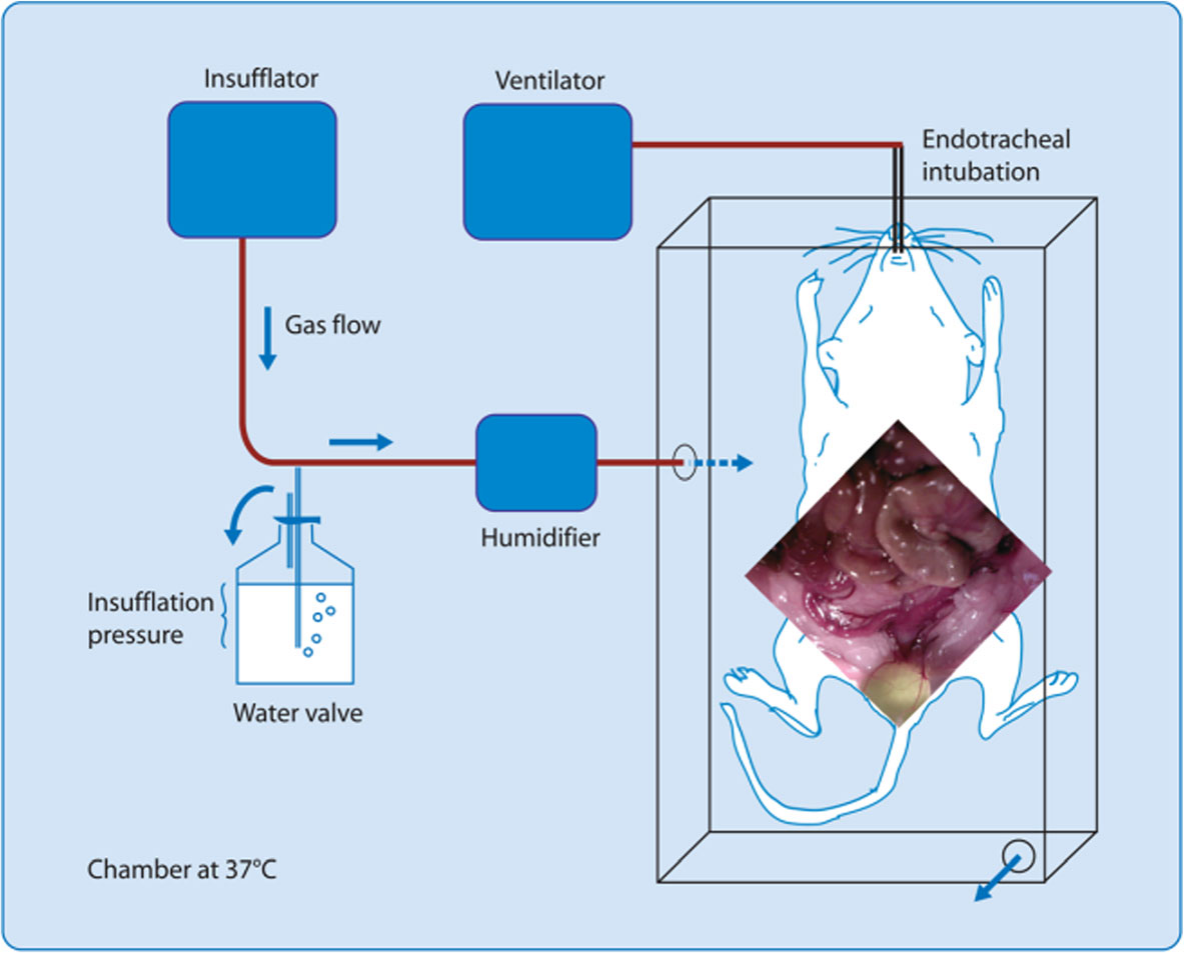
The mouse model for open surgery. Image modified from Binda et al. [13], Corona R et al, Gynecol Surg, 2017 + ref

Following some pilot experiments, the model was standardised as follows. Following anaesthesia (*T*_0_), shaving and disinfection of the abdomen, the mouse was placed on a warm pillow in a transparent plexiglas box measuring 22 ×10 ×30 cm, closed with a sliding transparent cover that could be removed to perform at 20 min, *T*_2_ surgery and lesions (Fig. 1). The box had one hole that accommodates the ventilation tube without gas leaks and two holes of 1 cm diameter each. The upper hole permitted escape of gas without pressure. The lower hole permitted insufflation of gas standardised in these experiments at 2 L/min. Since densities of CO_2_ and N_2_O are higher than air (*δ*CO_2_ = 1.842, *δ*N_2_O = 1.872, *δ*_air_ = 1.205 at room temperature and atmospheric pressure), the box fills progressively until the gas escapes by overflow. In order to perform the surgical procedure, the box had to be opened; this causes the insufflated gas to partially mix with the ambient air. A midline xyphopubic incision was performed, and the abdomen kept open with two pins. Standard 10× 2 mm bipolar lesions were created similar to the laparoscopic model. Following surgery, the cover was placed over the box and the mouse kept with the abdomen open exposed to the insufflated gas. This cover was necessary, since otherwise the insufflated gas would mix partially with the ambient air varying with the height of the box, the diameter of the opening and the flow rate of the gas insufflated. At the end of the experiment, the abdomen was closed with nylon 3-0 sutures.

### Scoring of adhesions

Postoperative adhesions were scored blindly after 7 days as previously described during a second laparotomy using a stereomicroscope. The terminology of Pouly et al. [15] was used to describe de novo adhesion formation as adhesions formed at non-surgical sites.

### Study design

#### Randomisation and factorial design

All experiments were block randomised by day as done in all previous experiments. Thus, one animal of each experimental group was operated at random on the same day in order to avoid eventual differences by day.

A factorial design [16] was used since a two by two factorial design results for each of the two variables in an almost similar statistical power as if two experiments had been performed with the same total number of animals in each experiment.

#### Mixture of N_2_O and CO_2_

In these experiments, we used either premixed gas with 90% CO_2_ and 10% N_2_O (Ijsfabriek, Strombeek, Belgium) or two Thermoflators one delivering CO_2_ and the other N_2_O, or the premixed gas. The gases from both insufflators were subsequently mixed in a mixing chamber, and the excess gas was permitted to escape from a water valve, the flow of both gases entering the box was limited to 2 L/min with a stopcock.

#### Pilot experiments

The first pilot experiment for open surgery consisted of mice (*n* = 3) with the abdomen open exposed to the ambient air at 37 °C (chamber at 37 °C) for 60 min. After 60 min, bowels were macroscopically dry and all mice died within 2 days. Mortality was thought to be caused by desiccation and maybe the damaging effect of 20% of O_2_ in air. Therefore, the box was designed as described in order to control the gas environment, and CO_2_ was used as a carrier gas in order to be comparable with laparoscopy and because CO_2_ is heavier than air and thus will fill the box progressively from the bottom.

A second pilot experiment was performed to evaluate in open surgery gas conditions known from the laparoscopy model. Humidified CO_2_ for 60 min confirmed the absence of mortality with humidification; humidified 50% CO_2_ + 50% N_2_O and humidified 100% N_2_O confirmed the strong adhesion preventing the effect of N_2_O in concentrations over 5% (six mice, two mice per group).

#### Experiment I

The first experiment was designed to evaluate in open surgery the effect of humidification and of the duration of exposure to either 100% CO_2_ or 100% N_2_O or 96% CO_2_ + 4%O_2_ upon adhesion formation. A factorial design was used with non-humidified or humidified gas (two factors), during 30 or 60 min (two factors), and the three gas compositions (three factors). With three mice/cell for two humidification factors and two duration factors and three gas factors (total mice = 3×2×2×3 = 36), an almost similar statistical power for each variable was obtained as if in three consecutive experiments with 36 mice each would have been done.

#### Experiment II

A dose response of the addition of various concentrations of N_2_O to the CO_2_ was evaluated in open surgery. Mice were exposed for 30 min to humidified CO_2_ with concentrations of N_2_O varying from 0 to 0.3, 1, 3, 10 and 100%. For 100% CO_2,_ 100% N_2_O and 10% N_2_O + 90% of CO_2_, a Thermoflator was used with CO_2_, N_2_O or a premixed gas (10% N_2_O + 90% CO_2_), respectively. For the other concentrations two Thermoflators were used, one with CO_2_ and the other with premixed gas (10% N_2_O + 90% CO_2_). The final concentrations of 3, 1, and 0.3% N_2_O were obtained by combining various flow rates of 4 and 2 L/min, 9 and 1 L/min and 14.5 and 0.5 L/min of 100% CO_2_ and premixed gas with 10% of N_2_O (six mice/group, total mice = 36).

#### Experiment III

The third experiment was designed in order to compare adhesion formation following laparoscopic and open surgery and to evaluate whether the addition of 4% of O_2_ had an additive effect when 10% of N_2_O had been added to the CO_2_. Using a factorial design, mice were exposed for 60 min to humidified 90% CO_2_ + 10% N_2_O or to 86% CO_2_ + 10% N_2_O + 4% O_2_ either during laparoscopy or during open surgery. Since adhesions were known to be very low with 10% of N_2_O, 10 mice per cell were used in order to have a power of almost 40 mice for each factor (total mice = 40).

### Statistics

Differences were calculated with the SAS System (SAS Institute, Cary, NC) [17] using Wilcoxon/Kruskal Wallis unpaired test for comparison of individual data and a two-way analysis of variance (Proc GLM) for experiments with a factorial design. Results are expressed as a mean and standard deviations unless indicated otherwise.

## Results

### Experiment I

**Fig. 2:**
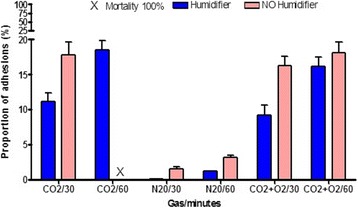
Effect of duration, humidification and gas type upon mortality and adhesion formation during open surgery: 30 or 60 min of non-humidified or humidified CO_2_, N_2_O or 96% CO_2_ + 4%O_2_ Corona R et al, Gynecol Surg, 2017 + ref were used. Proportions of adhesions are given (mean and SD)

As observed in the pilot experiment for open surgery with non-humidified air for 60 minutes, open surgery with non-humidified CO_2_ for 60 min resulted in 100% mortality (3/3). Even exposure to 30 min non-humidified CO_2_ resulted in 33.3% mortality (1/3) (Fig. 2). As expected, there was no mortality when humidified gas was used. To our surprise, there was also no mortality when 10% of N_2_O or 4% of O_2_ were added to the non-humidified CO_2_.

Adhesions at the surgical lesion site increased when the duration of exposure was longer (*p* < 0.0001) and when non-humidified gas was used (*p* < 0.0001) When 100% N_2_O was used, adhesions were very scant in comparison with 100% CO_2_ and 96% CO_2_ + 4%O_2_ with and without humidification (all comparisons *p* < 0.0001). When 96% CO_2_ + 4% O_2_ was used, adhesions were slightly less than with 100% CO_2_ (non-humidified gas for 30 min *p* < 0.0001; humidified gas for 30 min *p* = 0.0011 and for 60 min *p* = 0.003).

Whereas in the laparoscopic mouse model, de novo adhesions in the upper abdomen have never been observed; in this experiment, de novo adhesions were seen in the upper abdomen between bowels, and between bowels and sidewalls when non-humidified CO_2_ or non-humidified 96% CO_2_ + 4%O_2_ were used (Fig. 2).

### Experiment II

**Fig. 3:**
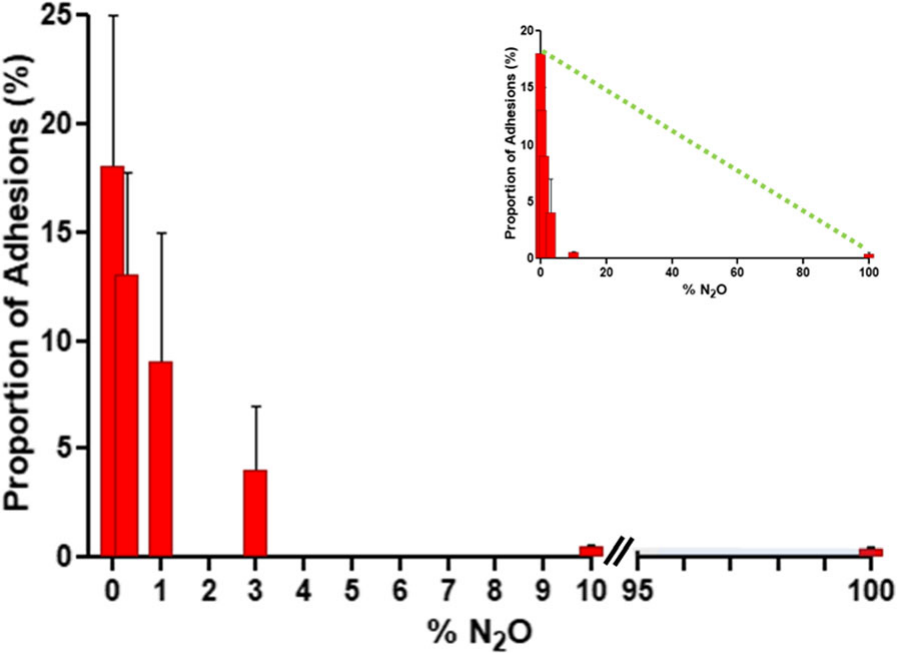
Dose-response curve of the addition of 0.3 to 100% N_2_O to humidified CO_2_ upon adhesion formation during open surgery demonstrating the drug-like effect. In inset, the dotted yellow line indicates adhesion formation if the effect of N_2_O would have been by replacing CO_2_ irritation. Proportions of adhesions are given (mean and SD) Corona R et al, Gynecol Surg, 2017 + ref

The addition of increasing concentrations of N_2_O to CO_2_ decreases exponentially adhesion formation, with a half maximum effect around 2.5% and a maximal effect from 5% onwards (Fig. 3). The difference between 10 and 100% N_2_O was not significant (*p* = 0.1551). Differences between 0 and 3%, 0 and 10% and between 3 and 10% N_2_O were *p* = 0.0061, *p* = 0.0006 and *p* = 0.03, respectively.

### Experiment III

**Fig. 4:**
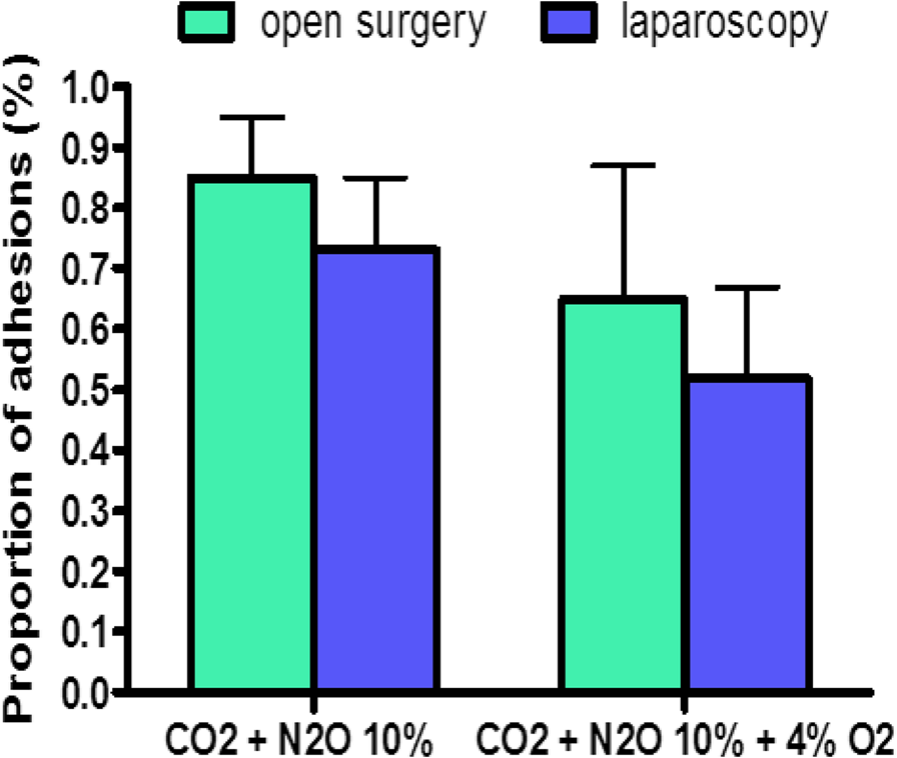
The additional effect of 4% of O_2_ on adhesion formation when 10% of N2O is used was investigated during laparoscopic and open surgery using humidified CO_2_. Adhesion formation was comparable between laparoscopic and open surgery. The additive effect of 4% of O_2_ was marginal (*p* < 0.01). Proportions of adhesions are given (mean and SD) Corona R et al, Gynecol Surg, 2017 + ref

When humidified CO_2_ with 10% of N_2_O was used, adhesion formation was as expected very low in all groups (Fig. 4). The extent of adhesions was similar after both laparoscopic and open surgery with (NS) or without (NS) 4% of oxygen. The addition of 4% of oxygen to CO_2_ with 10% of N_2_O had a very small additive effect which however turned out to be significant (*p* < 0.01) caused by the high power of 20 mice in each group (factorial design, 10 mice/cell) and the low variability of inbred strains,

## Discussion

These experiments confirm that the prevention of adhesion formation by conditioning is similar in both open and laparoscopic surgery. The main damaging effect of CO_2_, thus, is caused by mesothelial cell hypoxia and retraction, and less by tissue and ischaemia-reperfusion. Adhesions indeed increase with the duration of exposure to CO_2_ and with desiccation. N_2_O in concentrations of more than 5% appears to be the single most effective factor with a marginal beneficial effect when 4% of oxygen is added to this gas mixture. Although not all observations made during laparoscopic surgery were repeated in open surgery, we conclude that the mechanisms involved are similar in both open and laparoscopic surgery. The key factors are mesothelial cell damage and acute inflammation in the entire peritoneal cavity, as a reaction to trauma, hypoxia, ROS, oxidative stress and desiccation.

The exact mechanisms involved in the peritoneal cavity that enhance or prevent adhesion formation are not fully understood. The half maximal effect around 2.5% of N_2_O indicates that N_2_O has a drug-like effect, the mechanism of which is unknown. It is also not understood why mortality is 100% after exposure for 60 min to non-humidified CO_2_ and no mortality when non-humidified N_2_O is used, although the desiccated aspect of the bowels is the same. We only can speculate that mortality is not only caused by the desiccation but mainly by the severity of the inflammatory process since N_2_O strongly and O_2_ slightly decrease the inflammatory reaction [18].

The observed effects of 5 to 10% of N_2_O in open surgery at atmospheric pressure shed new light on the pathophysiology of adhesion formation. CO_2_ pneumoperitoneum at an insufflation pressure of 15 mmHg decreases peritoneal oxygenation, triggers hypoxemia inducible factor (HIF) and decreases tissue plasminogen activator and upregulates PAI for several days. These effects of CO_2_ pneumoperitoneum are less and/or of shorter duration at lower insufflation pressures and disappear at insufflation pressures below 8 mmHg in human and 2 mmHg in mice [3, 6–8]. Taking into account the differences in size between man and mice and Pascal’s law, these pressures are considered the pressures at which vascular compression of the peritoneum, hypoxia and oxidative stress start. Since at atmospheric pressure N_2_O still decreases adhesion formation caused by the CO_2_ environment, we must conclude that key mechanism driving the subsequent events is mesothelial hypoxia and retraction. In addition, the observations that with laparoscopic surgery in the presence of a 10% N_2_O environment at 15 mmHg pressure, the extent of adhesion formation is similar to open surgery at atmospheric pressure strongly suggests that 10% of N_2_O prevents mesothelial cell oxidative stress hypoxia and its consequences including mesothelial cell retraction and decreased fibrinolysis enhanced adhesion formation and postoperative pain. However, whether N_2_O also has a protective effect on oxidative stress caused by partial oxygen pressures higher than 75 mmHg (or more than 10% O_2_ at atmospheric pressure) as in air remains to be investigated.

Despite the differences that exist between oxidative stress caused by 20% CO_2_ in ambient air in open surgery and the detrimental effect of the CO_2_ pneumoperitoneum and insufflation pressure, the prevention of adhesion formation and postoperative pain are similar in both open and laparoscopic surgery. Beside the use of a proper atraumatic surgical technique and precise haemostasis, the important adhesion preventive factors in open surgery are to avoid ROS formation, caused by the 20% oxygen concentration in ambient air; the use of N_2_O in concentrations of 5% or more; cooling the peritoneal cavity; avoiding desiccation; the use of Ringer’s lactate solution instead of saline; being toxic for mesothelial cells [19–25] for intraoperative irrigation and terminal thorough lavage and administration of one or two doses of dexamethasone after surgery. Although, it has not been investigated as yet whether N_2_O can prevent the damaging effects of exposure to 20% of oxygen concentration, the flooding of the surgical site in open surgery with 5 to 10% of N_2_O will require a carrier gas for which both CO_2_ and nitrogen (N_2_) seem suitable. The importance of cooling in open surgery has indirectly been confirmed in rats using cold saline infusions [26, 27]. Adhesions were also less when the abdominal cavity was exposed to the atmosphere of the operating theatre (21% O_2_, 21 °C, 40–47% relative humidity) than to CO_2_ + 4% of oxygen and 95–100% relative humidity at 37 °C [28]. Prevention of desiccation is much more important in open surgery than in laparoscopic surgery considering the 100% mortality of mice when exposed to dry CO_2_ for 60 min. The toxicity of saline for the peritoneum was known since the early 1970s [19, 20] and has recently been confirmed [21–25]. The use of dexamethasone and another tenet of microsurgery was only proven to be effective after conditioning in laparoscopic surgery in an animal model. In any case, adhesion formation following open and laparoscopic surgery appears to be remarkably similar in an atmosphere of 10% N_2_O in CO_2_ without desiccation.

The implementation of these principles to open surgery should be carried out judiciously. That saline should be abandoned, and a richer solution should be used for irrigation is obvious. Prevention of desiccation can be achieved by continuous irrigation as done in microsurgery, by covering bowels with moistened inert towels and/or by flooding the operative field with humidified CO_2_ [29, 30]. The latter indeed decreased adhesion formation in open cardiac surgery. The instillation of humidified CO_2_ deep into the surgical field also decreased oxidative stress since the organs were no longer exposed to the 20% of O_2_ in ambient air. A similar effect was achieved by shielding the organs in microsurgery. The exposure of the surgical field to the temperature of the operating theatre has never been an issue in open surgery. We can be happy today that cooling unexpectedly has a beneficial effect. The administration of dexamethasone after surgery, eventually at the end of surgery, may be beneficial to reduce inflammation and adhesion formation and accelerate recovery, while its use might aggravate an eventual infection. The proven very strong beneficial effect of 5 to 10% of N_2_O with no explosion risk demands a trial in open surgery. As described for humidified CO_2_ in cardiac surgery, [29, 30] the deep instillation of gases heavier than air will fill and flood progressively the operation field. CO_2_ seems obvious as a carrier gas since it is heavier than air with minimal irritative effect at atmospheric pressure. N_2_O, which fortunately also is heavier than air, should be used in concentrations of 5 to 10% of N_2_O. For this reason, we used the same combination for these experiments, the efficacy of which had furthermore already been proven in animal models. It will obviously be necessary to prevent or reduce contamination of the operating theatre with N_2_O. The suggested upper threshold for N_2_O is 25 ppm [31]. We speculate that this can be achieved with aquarium-like drapings extending above the operating field with aspiration at the borders to prevent overflow; the opening of the draping would be a compromise between being sufficiently large to permit surgery but small enough to prevent mixture with the ambient air. Indeed even without aspiration contamination with 2 L/min with 10% of N_2_O would result in only 15 ppm N_2_O in a normal sized (e.g. 40 m^3^) and ventilated (e.g. refresh rate of 20 cycles/h) operating room.

## Conclusions

In conclusion, the mechanisms of mesothelial cell damage and their prevention are the same with open and with laparoscopic surgery. The application of microsurgical tenets, which enabled to decrease inflammation in the peritoneal cavity, reduce adhesion formation and improve fertility outcomes, can benefit further from flooding the operative field with 5 to 10% of N_2_O, which has proven to be a most effective factor. Prevention of mesothelial cell damage and the subsequent acute inflammation may even be more important in open surgery than in laparoscopic surgery, especially when bowels are exteriorised and subjected to desiccation and exposed to ambient air that creates oxidative stress. Since both CO_2_ and N_2_O are heavier than air, it is also possible to instil humidified CO_2_ with 5 to 10% of N_2_O deep into the abdominal cavity during the procedure. Given the absence of side effects, as demonstrated in laparoscopic surgery, a study in open surgery may well demonstrate a virtually adhesion free surgery, a reduction of postoperative pain and a shortened recovery period, as has been observed in laparoscopic surgery.
